# Genome-wide analysis of lncRNAs, miRNAs, and mRNAs forming a prognostic scoring system in esophageal squamous cell carcinoma

**DOI:** 10.7717/peerj.8368

**Published:** 2020-02-10

**Authors:** Xiaobo Shi, You Li, Yuchen Sun, Xu Zhao, Xuanzi Sun, Tuotuo Gong, Zhinan Liang, Yuan Ma, Xiaozhi Zhang

**Affiliations:** 1 Department of Radiation Oncology, The First Affiliated Hospital of Xi’an Jiaotong University, Xi’an, Shaanxi, China; 2 Department of Peripheral Vascular Diseases, The First Affiliated Hospital of Xi’an Jiaotong University, Xi’an, Shaanxi, China

**Keywords:** Esophageal squamous cell carcinoma, lncRNA, miRNA, mRNA, Prognostic scoring system, prognosis-related co-expression network

## Abstract

**Background:**

Esophageal squamous cell carcinoma (ESCC) is the main subtype of esophageal carcinoma. Protein coding genes and non-coding RNAs can be powerful prognostic factors in multiple cancers, including ESCC. However, there is currently no model that integrates multiple types of RNA expression signatures to predict clinical outcomes.

**Methods:**

The sequencing data (RNA-sequencing and miRNA-sequencing) and clinical data of ESCC patients were obtained from The Cancer Genome Atlas (TCGA) database, and Differential gene expression analysis, Cox regression analysis and Spearman correlation analysis were used to construct prognosis-related lncRNA-mRNA co-expression network and scoring system with multiple types of RNA. The potential molecular mechanisms of prognostic mRNAs were explored by functional enrichment analysis.

**Results:**

A total of 62 prognostic lncRNAs, eight prognostic miRNAs and 66 prognostic mRNAs were identified in ESCC (*P*-value < 0.05) and a prognosis-related lncRNA-mRNA co-expression network was created. Five prognosis-related hub RNAs (CDCA2, MTBP, CENPE, PBK, AL033384.1) were identified. Biological process analysis revealed that mRNAs in prognosis-related co-expression RNA network were mainly enriched in cell cycle, mitotic cell cycle and nuclear division. Additionally, we constructed a prognostic scoring system for ESCC using ten signature RNAs (MLIP, TNFSF10, SIK2, LINC01068, LINC00601, TTTY14, AC084262.1, LINC01415, miR-5699-3p, miR-552-5p). Using this system, patients in the low-risk group had better long-term survival than those in the high-risk group (log-rank, *P*-value < 0.0001). The area under the ROC curve (AUCs) revealed that the accuracy of the prediction model was higher than the accuracy of single type of RNA prediction model.

**Conclusion:**

In brief, we constructed a prognostic scoring system based on multiple types of RNA for ESCC that showed high predicting prognosis performance, and deeply understood the regulatory mechanism of prognosis-related lncRNA-mRNA co-expression network.

## Introduction

Esophageal cancer is one of the most common malignancies, ranking 7th in global morbidity and 6th in cancer-related mortality (Bray et al., 2018). The pathological types of esophageal cancer are mainly squamous cell carcinoma and adenocarcinoma. Esophageal squamous cell carcinoma (ESCC) is prevalent in Asia, Africa, and South America, especially in China, where ESCC accounts for more than 90% (Malhotra et al., 2017). The main treatments for esophageal cancer include surgical resection, radiotherapy and chemotherapy. Although progress has been made in the diagnosis and treatment of esophageal cancer, the 5-year overall survival rate is only about 15–20% (Chen et al., 2016; Fitzmaurice et al., 2015; Gavin et al., 2012). At present, the gold standard of tumor treatment and survival prediction is still tumor node metastasis (TNM) staging system, but there are some limitations in clinical application (Amin et al., 2017). TNM staging can only include categorical variables such as tumor, lymph node or metastasis, while neglecting other important prognostic variables, such as genomics or transcriptome differences. TNM staging also is difficult to explain why patients of the same stage have different clinical outcomes after the same treatment, that is, it cannot distinguish individual differences in patients with the same stage. Therefore, it is necessary to establish a genome or transcriptome based prognostic score system to predict the clinical prognosis of individual patients more accurately.

According to some estimates, about 70% of the human genome is transcribed into RNA, the portion of the genome which codes for proteins is only about 2% (Birney et al., 2007; Esteller, 2011). In recent decades, protein coding genes and non-coding RNAs have been confirmed to play key roles in tumorigenesis and tumor progression. For ESCC, researchers have identified multiple driving genes, including TP53, NOTCH1, FAM135B, EP300, and TET2, and the mutation status of FAM135B, EP300 and TET2 are associated with the prognosis of patients (Gao et al., 2014; Sawada et al., 2016; Song et al., 2014). Wen et al. (2019) analyzed the expression profile of small non-coding RNAs in 145 ESCC samples, and established a prediction model composed of four-miRNAs, which was used to predict overall survival in LN-positive locoregional ESCC patients. Sun et al. (2015) analyzed the expression of GASC1-targeted gene in 149 tumor specimens from patients with ESCC, and identified a prediction model composed of three-gene (PPARG, MDM2, and NANOG), which may serve as a predictor for the poor prognosis of ESCC patients. Li et al. (2016) conducted whole-genome sequencing analysis of lncRNA expression in 12 ESCC tumor and normal tissues, and constructed a co-expression network composed of 119 differentially expressed lncRNA and 1350 correlated mRNAs to reveal the potential mechanism of ESCC. However, the individualized prognosis prediction model based on multiple types of RNA has not been reported in ESCC, and the prognosis-related lncRNA-mRNA co-expression network is lacking.

In this study, we comprehensively analyzed the expression and clinical data of lncRNAs, miRNAs and mRNAs of ESCC in the TCGA database. Using multivariate Cox regression analysis, we constructed a prognostic scoring system based on multiple types of RNA that divided ESCC patients into two groups (high-risk and low-risk) with a significant difference in overall survival (OS). The accuracy of the prognostic scoring system was higher than the accuracy of single type of RNA prediction model. Besides, we constructed a prognosis-related lncRNA-mRNA co-expression network in ESCC and the potential molecular mechanisms of prognostic mRNAs were explored by functional enrichment analyses. The presented analysis, we aim to provide novel clues for effective prediction of clinical outcomes.

## Material and Methods

### Data collection and pretreatment

The sequencing data (RNA-sequencing and miRNA-sequencing) and clinical information of ESCC patients were obtained from the TCGA database (https://portal.gdc.cancer.gov/). Based on the annotation file (Homo_sapiens.GRCh38.95.chr.gtf) downloaded from the Ensembl database (http://asia.ensembl.org/info/data/ftp/index.html), we identified 19876 lncRNAs and 19645 protein-coding genes. At the same time, we identified 2069 miRNA according to the annotation file (mature.fa) downloaded from miRBase database (http://mirbase.org/ftp.shtml). LncRNAs, mRNAs and miRNAs expressing raw count value >1 were screened for subsequent operation. This study was in line with the published guidelines provided by TCGA (https://cancergenome.nih.gov/publications/publicationguidelines). Since our data was obtained from the TCGA database, no ethics committee approval was required.

### Differentially expressed analysis

The analysis and extraction of differentially expressed lncRNAs and mRNAs between 81 tumor tissues and 11 normal tissues were conducted by using the edgeR package of R language (Robinson, McCarthy & Smyth, 2010; R Core Team, 2013). Similarly, the differentially expressed miRNAs between 95 tumor tissues and 13 normal tissues were analyzed and extracted using edgeR package. |log2FC| > 2 and FDR < 0.05 (FC, fold change; FDR, false discovery rate) were considered to be significant. After edgeR normalization, log2 (normalized value +1) transformation was performed on the expression profiles of miRNAs, mRNAs and lncRNAs for subsequent manipulation.

### Survival analysis

The clinical datasets of the ESCC cohort were downloaded from TCGA. Samples with a survival time of *t* = 0 days were removed to avoid introducing more mixed factors, and the remaining 80 samples were retained for the survival analysis. The clinical and pathological characteristics of the remaining 80 samples are summarized in Table 1. Univariate Cox regression analysis was used in R software to evaluate whether lncRNA, miRNA and mRNA were correlated with OS. RNAs with *P* < 0.05 were screened as prognostic biomarkers. RNAs with hazard ratio (HR) <1 were defined as protective signature, while RNAs with HR for death >1 were defined as risky RNAs.

**Table 1:** Clinical characteristics of 80 patients with esophageal squamous cell carcinoma.

**Characteristics**	**Number**	**Percent (%)**
Gender		
Male	68	85
Female	12	15
Age (years)		
≤58	46	57.5
>58	34	42.5
Histologic grade		
G1	15	18.75
G2	38	47.5
G3	18	22.5
G_X_	9	11.25
Tumor stage		
T1	7	8.75
T2	29	36.25
T3	40	50
T4	4	5
Node stage		
N0	42	52.5
N1 + N2	30	37.5
N_X_	8	10
Metastasis stage		
M0	70	87.5
M1	5	6.25
M_X_	5	6.25
Pathologic stage		
I	7	8.75
II	46	57.5
III	22	27.5
IV	4	5
–	1	1.25
Survival status		
No	24	30
Yes	56	70

### LncRNA-mRNA co-expression network

The correlation between prognostic lncRNA and mRNA expression profiles was analyzed by Spearman method, and the lncRNA-mRNAs pairs that the absolute value of correlation coefficients > =0.4 and *p* < 0.05 were selected to construct the co-expression network. The co-expression network result was displayed using Cytoscape software version 3.6.0 (https://cytoscape.org/) (Shannon, 2003). CytoHubba, a plugin in the Cytoscape software, was adopted to calculate the degree of each node and select modules of hub genes from the network (Chin et al., 2014).

### Functional enrichment analysis

Gene Ontology (GO) and Kyoto Encyclopedia of Genes and Genomes (KEGG) functional and pathway enrichment analysis were performed for mRNAs in prognosis-related co-expression RNA network using the Database for Annotation, Visualization and Integrated Discovery bioinformatics resources (DAVID) (https://david-d.ncifcrf.gov/), and *P* < 0.05 was considered as the cut-off criterion to screen the Enriched terms and pathways (Altermann & Klaenhammer, 2005; Ashburner et al., 2000; Huang, Sherman & Lempicki, 2009a; Huang, Sherman & Lempicki, 2009b).

### Prognostic scoring system

**Figure 1:**
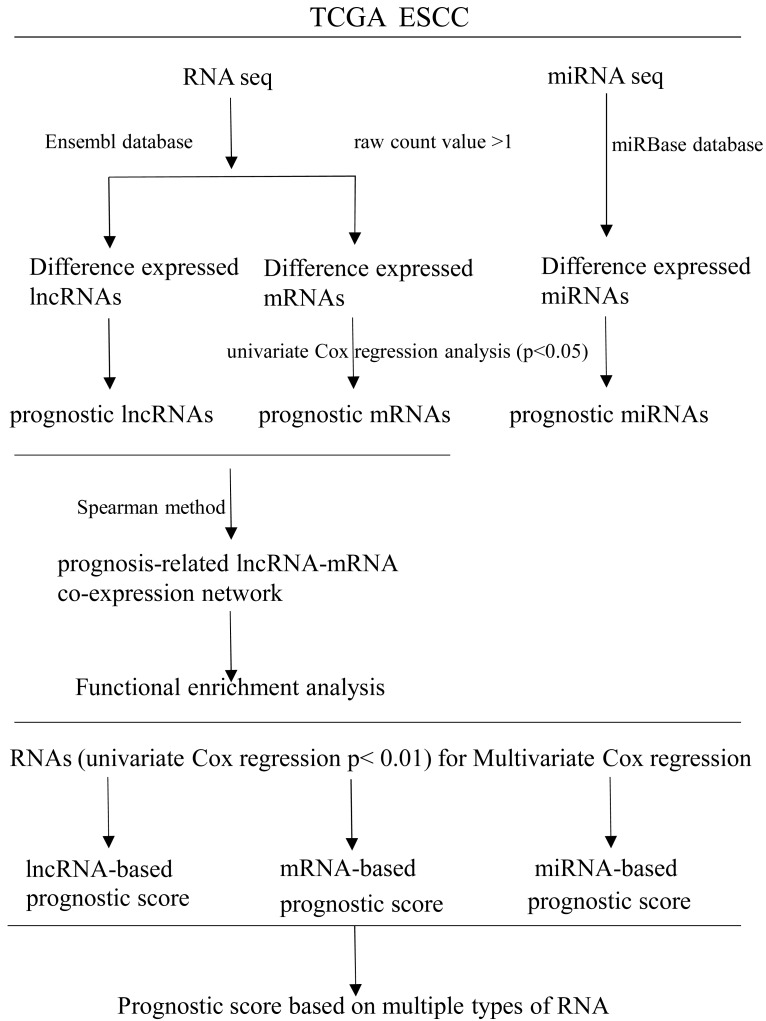
Flow diagram of the prognostic scoring system construction.

RNAs with univariate Cox regression *P* < 0.01 were selected for the stepwise Cox regression procedures. Akaike information criterion (AIC) was used to evaluate the relative goodness of fitted model. Furthermore, Multivariate Cox regression coefficient was multiplied by the expression level of independent biomarkers (*P* < 0.05) to construct prognostic Cox models of lncRNA, miRNA and mRNA, respectively. Finally, a prognostic scoring system in ESCC was constructed, based on above-described multiple types of RNA. Receiver operating characteristics curves (ROC) and area under ROC curves (AUCs) were applied to evaluate the efficiency of each model. Statistical computing was performed using R software version 3.5.2. A flow diagram of the prognostic scoring system is presented in Fig. 1.

### Statistical analysis

The statistical analyses in the present study were conducted by SPSS Statistics 18.0 and R software version 3.5.2. *P* value <0.05 was defined as statistically significance. Univariate Cox and multivariate Cox regression analyses were used to identify prognostic biomarkers. Survival curves were plotted by Kaplan–Meier (K-M) analysis, and differences in survival rates were assessed using a log-rank test.

## Results

### Differentially expressed lncRNA, miRNA and mRNA

**Figure 2:**
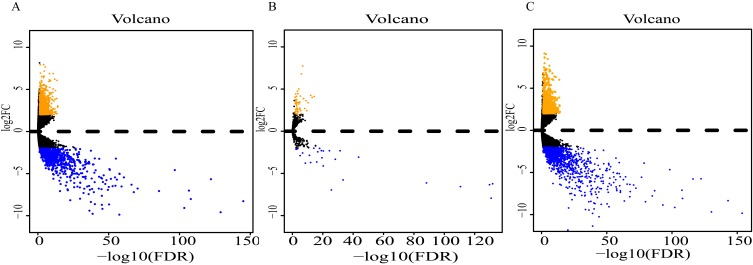
Volcano plot of differentially expressed RNAs between ESCC and normal tissues. (A) lncRNAs; (B)miRNAs; (C) mRNAs. Orange dots indicate upregulated RNAs, while blue dots indicate downregulated RNAs with statistical significance.

Analysis of expression profiles in ESCC compared with normal esophageal tissues identified a total of 1662 lncRNAs, 79 miRNAs and 2063 mRNAs (Table S1). Among them, 818 and 844 lncRNAs were respectively up-regulated and down-regulated (Fig. 2A); 52 miRNAs were up-regulated, and 27 were down-regulated (Fig. 2B); 869 up-regulated mRNAs and 1196 down-regulated mRNAs were obtained (Fig. 2C). Expression heatmaps were constructed by the top 50 up-regulation and the top 50 down-regulation to visualize the most significant lncRNAs, miRNAs and mRNAs (Fig. S1). The heatmap of the lncRNAs (Fig. S1A), miRNAs (Fig. S1B) and mRNAs (Fig. S1C) showed that the tumors clustered separately from the normal tissues.

### Prognostic lncRNAs, miRNAs, mRNAs and co-expression network

**Figure 3:**
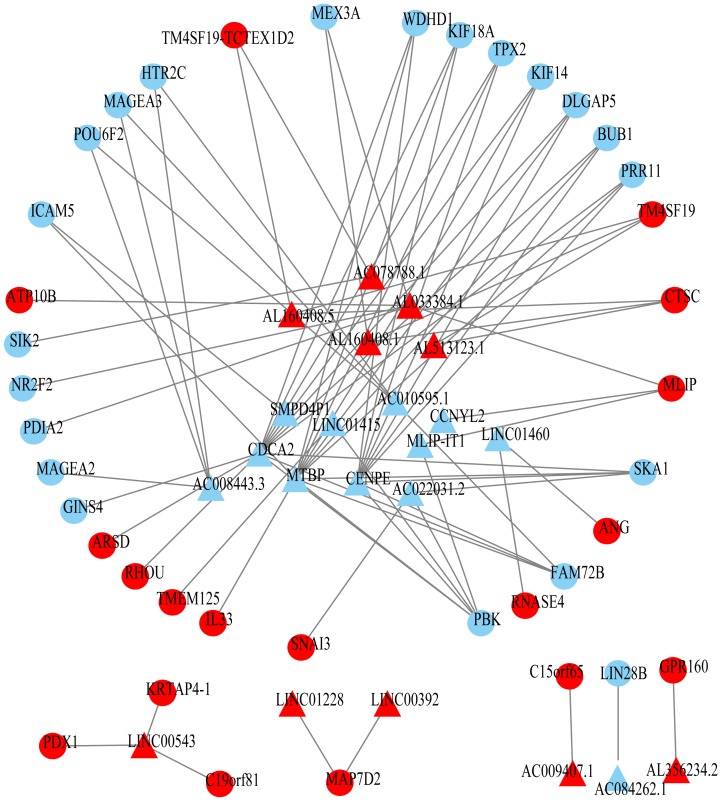
Prognosis-related co-expression RNA network in ESCC. The red represents the risky RNAs, and the blue represents the protective RNAs in ESCC. The triangle indicates lncRNAs and circle indicates mRNAs.

**Figure 4:**
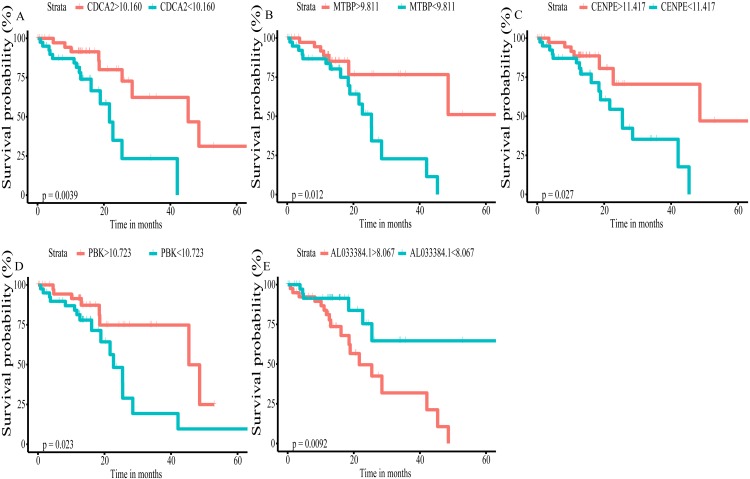
Kaplan–Meier (K–M) survival curves for top5 RNAs in the prognosis-related co-expression RNA network. (A) CDCA2; (B) MTBP; (C)CENPE; (D) PBK; (E) AL033384.1.

Using univariate Cox regression analysis on the remaining 80 samples with survival times >0, 62 prognostic lncRNAs, eight prognostic miRNAs, and 66 prognostic mRNAs were identified in ESCC (*P*-value <0.05) (Table S2). The prognostic lncRNAs and mRNAs in ESCC were used to generate the co-expression network consisting of 22 lncRNAs, 40 mRNAs, and 77 interaction pairs (Fig. 3) (Table S3). Cytoscape analysis of the co-expression network revealed the top five prognostic RNAs (CDCA2, MTBP, CENPE, PBK, AL033384.1) (Table 2). Based on the median expression of each top 5 RNAs, 80 ESCC patients were divided into two groups (high expression vs low expression). The prognostic value of RNA was demonstrated by K-M plots (Fig. 4).

**Table 2:** Top five in the prognosis-related co-expression RNA network ranked by degree method.

**Rank**	**Name**	**Score**	**HR**
1	CDCA2	13	0.508863
2	MTBP	12	0.566911
3	CENPE	10	0.63653
4	PBK	6	0.627698
5	AL033384.1	5	1.460057

**Figure 5:**
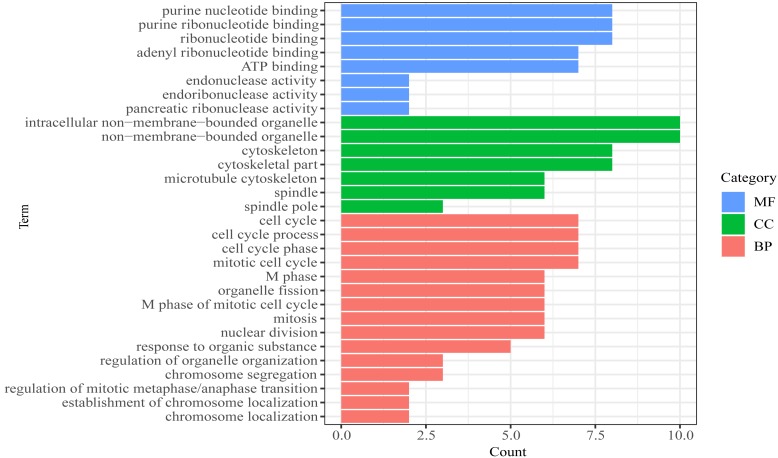
Gene Ontology (GO) analysis of mRNA in the prognosis-related co-expression RNA network.

We then performed GO functional enrichment analysis of mRNAs in prognosis-related co-expression RNA network (Fig. 5). The results showed that the prognostic mRNAs mainly enriched in biological process (BP) including cell cycle, mitotic cell cycle and nuclear division. Cellular component (CC) analysis indicated enrichment in intracellular non-membrane-bounded organelle, non-membrane-bounded organelle and cytoskeletal part. Besides, in the molecular function (MF), the mRNAs were significantly clustered into purine nucleotide binding, ribonucleotide binding and ATP binding terms (Table S4). No pathways were significantly enriched in the KEGG enrichment analysis of prognostic mRNAs.

### Prognostic scoring system

**Figure 6:**
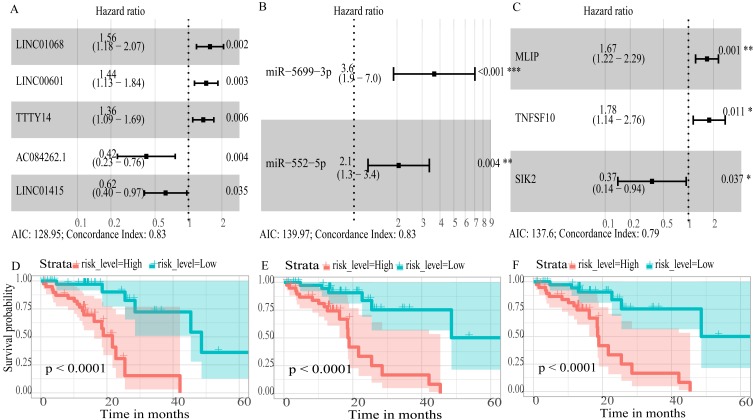
Forest plots of hazard ratios (HR) of the RNAs and Kaplan-Meier curves for overall survival (OS) of high-risk and low-risk patients based on prognostic scores in the TCGA ESCC cohort. (A) lncRNAs; (B)miRNAs; (C) mRNAs; (D) lncRNA-based prognostic score; (E) miRNA-based prognostic score; (F) mRNA-based prognostic score.

To create prognostic scoring system, RNAs with univariate Cox regression *P* < 0.01 were selected for the stepwise Cox regression procedures. Next, based on 5 lncRNAs, 2 miRNAs and 3 mRNAs respectively, we constructed three prediction models with single type of RNA to calculated the risk scores for predicted survival (Table S5). The formulas for the three prognostic models were as follows: lncRNA-based prognostic score = (0.447 × expression level of LINC01068) + (0.3677 × expression level of LINC00601) + (0.3075 × expression level of TTTY14) + (−0.8750 × expression level of AC084262.1) + (−0.4744 × expression level of LINC01415); miRNA-based prognostic score = (1.2932 × expression level of miR-5699-3p) + (0.7202 × expression level of miR-552-5p); mRNA-based prognostic score = (0.5139 × expression level of MLIP) + (0.5746 × expression level of TNFSF10) + (−1.0069 × expression level of SIK2). Of three prognostic models, seven RNAs were shown to be risky RNAs (LINC01068, LINC00601, TTTY14, miR-5699-3p, miR-552-5p, MLIP, TNFSF10, HR >1) and three RNAs were the protective RNAs (AC084262.1, LINC01415, SIK2, HR <1) (Figs. 6A–6C).

**Figure 7:**
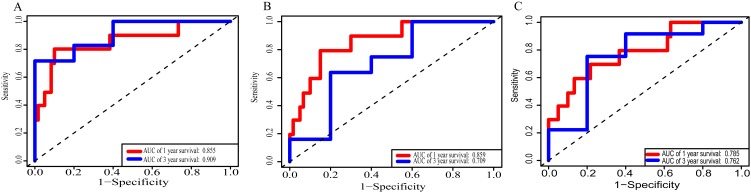
Receiver operating characteristic curves for survival prediction by prognostic score in TCGA ESCC cohort. (A) lncRNA-based prognostic score; (B) miRNA-based prognostic score; (C) mRNA-based prognostic score. The red indicates the AUC of 1 years survival and the blue indicates the AUC of 3 years survival.

**Figure 8:**
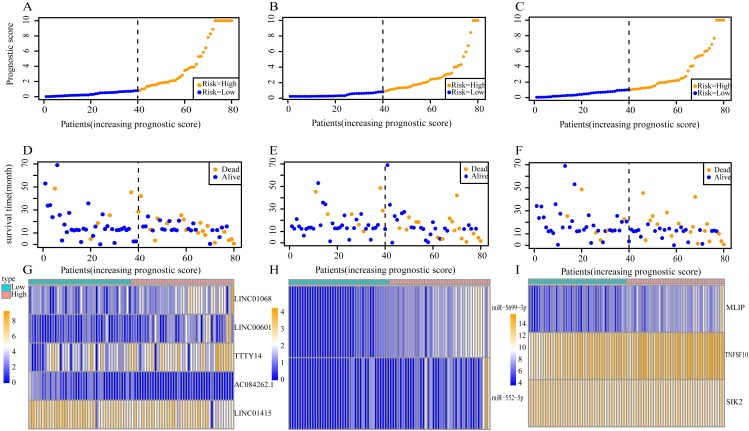
Prognostic scores, survival and expression clustering heatmap of the signature RNAs of ESCC patients. (A) Distribution of prognostic score based on lncRNA. (B) Distribution of prognostic score based on miRNA. (C) Distribution of prognostic score based on mRNA. (D) Distribution of patient survival status based on lncRNA prognostic scores. (E) Distribution of patient survival status based on miRNA prognostic scores. (F) Distribution of patient survival status based on mRNA prognostic scores. (G–I) Expression clustering heatmap of the signature RNAs.

Using these three formulas, we calculated the prognostic score for each of the 80 patients separately and ranked them according to the increased prognostic scores. we divided the ESCC patients into two group (high-risk or low-risk) using the median prognostic score as a cutoff. As shown in Figs. 6D–6F, patients in the high-risk group had a worse prognosis than the low-risk group in all three models (*P* < 0.0001). We also used ROC curves to estimate the specificity and sensitivity of these prognostic models. All three prognostic models showed moderate prognostic evaluation ability, with AUC of 1 year values of 0.855, 0.859 and 0.785, separately, and AUC of 3 year values of 0.909, 0.709 and 0.762, separately (Fig. 7). Figure 8 shows the distribution of patient prognostic scores, the survival status and tumor RNAs expression of all 80 ESCC patients. Patients in the high-risk group had more deaths than those in the low-risk group in all three models (Figs. 8D–8F). Moreover, patients in the low-risk group tend to express protective RNA, while patients in the high-risk group tend to express risky RNA (Figs. 8G–8I).

**Figure 9:**
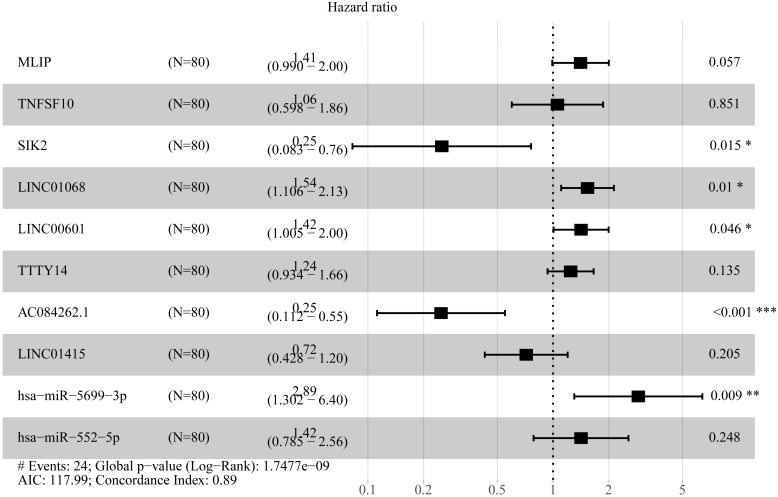
Forest plots of hazard ratios (HR) of the RNAs involved in prognostic scoring system based on multiple types of RNA.

**Figure 10:**
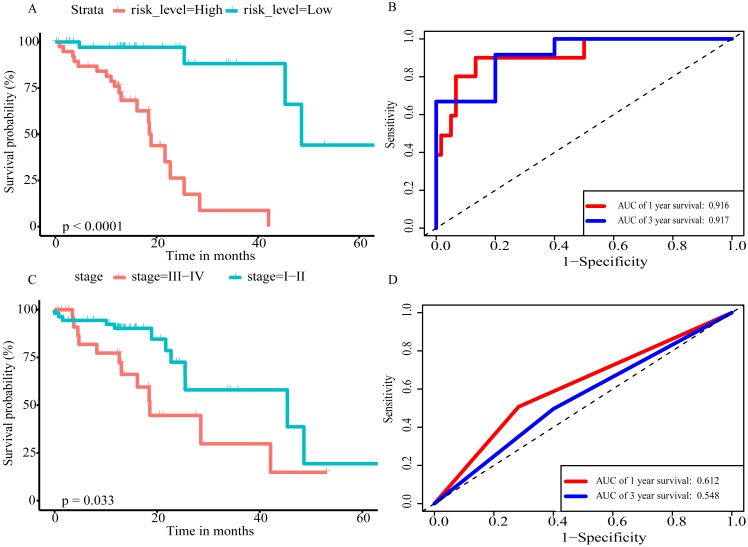
The prognostic value of prognostic score and TNM stage in the TCGA datasets. (A) Overall survival (OS) outcomes for high-risk and low-risk patients grouped by prognostic scoring system. (B) ROC curve with AUC of prognostic scoring system. (C) K–M plots of patients with different TNM staging. (D) ROC curve with AUC of TNM stage.

In order to improve the prediction accuracy and understand the potential molecular mechanism of prognostic markers, we constructed a prognostic Cox model with multiple types of RNA for ESCC using the ten RNAs provided above (Table S6). The formula was as follows: RNA-based prognostic score = (0.42895 × expression level of LINC01068) + (0.34829 × expression level of LINC00601) + (0.2185 × expression level of TTTY14) + (−1.393 × expression level of AC084262.1) + (−0.33364 × expression level of LINC01415) + (1.06024 × expression level of miR-5699-3p) + (0.34784 × expression level of miR-552-5p) + (0.3418 × expression level of MLIP) + (0.05437 × expression level of TNFSF10) + (−1.38365 × expression level of SIK2). The ten RNAs forest plots of RNA-based prognostic model was presented in Fig. 9. K-M analysis showed patients in the low-risk group had better long-term survival than those in the high-risk group (*P*-value <0.0001; Fig. 10A). Furthermore, the AUC of 1 year value was 0.916 and 3 year value was 0.917 (Fig. 10B), indicating that the combination of different types of RNA patterns is a more accurate prognostic model than single type of RNA prediction model. For tumor staging, we also generated K-M plots and corresponding ROC curves. ESCC patients were also divided into two groups by tumor, node and metastasis (TNM) stage, and the prognosis of the two groups was different (*P*-value <0.05; Fig. 10C). The AUC of 1 year value and 3 year value based on TNM staging were 0.612 and 0.548, respectively (Fig. 10D). Although TNM staging is often used in clinical prognostic prediction, its prognostic AUC value is limited. Besides, combining multiple clinical parameters, we performed cox regression analysis of the prognostic score. As shown in Table 3, in both univariate Cox and multivariate Cox regression analysis, prognostic score was significantly correlated with survival (*P* < 0.001), that is, prognostic score was an independent prognostic factor in ESCC patients.

## Discussion

ESCC is one of the leading causes of cancer-associated mortality worldwide. Several studies have shown that lncRNAs, miRNAs, and mRNAs can be powerful prognostic factors in multiple cancers, including ESCC. MALAT1 has been identified as an important predictor of survival in ESCC (Hu et al., 2015). Luo & Wu (2019) verified that miR-375 may be a new prognostic marker of ESCC by meta-analysis. Pan et al. (2014) measured FOXCUT/FOXC1 in 82 ESCC tissues and adjacent noncancerous tissues by real-time quantitative PCR (qPCR), and found patients with upregulated FOXCUT or FOXC1 experienced a significantly worse prognosis than those with downregulated FOXCUT or FOXC1. However, the prediction models constructed in the previous studies mainly focus on one kind of RNA, which has limited prognostic efficacy.

In the present study, we comprehensively analyzed the expression data and clinical data of ESCC in the TCGA database, and identified 62 prognostic lncRNAs, 8 prognostic miRNAs, and 66 prognostic mRNAs. Using Cox regression analysis, We proposed three different prognostic models based on 5 lncRNAs, 2 miRNAs and 3 mRNAs respectively, which showed moderate prognostic assessment ability in predicting long-term survival of ESCC patients. Furthermore, a novel prognostic scoring system that included multiple types of RNA was proposed, which showed high predicting prognosis performance and was validated as an independent prognostic factor in ESCC patients. Of prognostic models, seven RNAs were shown to be risky RNAs (LINC01068, LINC00601, TTTY14, miR-5699-3p, miR-552-5p, MLIP, TNFSF10, HR >1) and three RNAs were the protective RNAs (AC084262.1, LINC01415, SIK2, HR <1).

A number of RNAs in the prognostic system used in the present study have been previously implicated in malignant tumors. TTTY14 (testis-specific transcript, Y-linked 14) was significantly correlated with overall survival for gastric cancer (GC) patients and oral squamous cell carcinoma (OSCC) patients and has been suggested to be involved in HPV (human papillomavirus)-Induced Oncogenesis (Cheng et al., 2019; Goedert et al., 2016; Li et al., 2017). miR-552-5p facilitates osteosarcoma cell proliferation and metastasis by targeting WIF1, which means miR-552-5p may become a new target for the treatment of osteosarcoma (Cai et al., 2019). TNFSF10 (TNF superfamily member 10), a cytokine that belongs to the tumor necrosis factor (TNF) ligand family,preferentially induces apoptosis in transformed and tumor cells, and TNFSF10 was significantly associated with overall survival in patients with liver cancer, breast cancer, non-small cell lung cancer and other tumors (Koç Erbaşoğlu et al., 2019; McCarthy, 2005; Piras-Straub et al., 2015). Frequent amplification of TNFSF10 was associated with the development and progression of esophageal cancer (Chen et al., 2008). SIK2 (salt inducible kinase 2) was a potential breast cancer suppressor, and compared with normal control, its expression level of breast cancer tissues and cell lines was reduced (Maxfield et al., 2016). However, functional studies of the other RNAs (LINC01068, LINC00601, AC084262.1, LINC01415, miR-5699-3p, MLIP) have not been reported in cancer research.

**Table 3:** Univariate and multivariate Cox regression analysis of overall survival in ESCC. Age, pathologic stage, tumor stage, histologic grade and prognostic score were continuous variable. Specifically, pathologic stage: I = 1, II = 2, III = 3, IV = 4; Tumor stage: T1 = 1,T2 = 2, T3 = 3, T4 = 4; Histologic grade: G1 = 1, G2 = 2, G3 = 3.

**Variables**	**Univariate analysis**		**Multivariate analysis**	
	**Hazard radio (95% CI)**	***P***	**Hazard radio (95% CI)**	***P***
Age	1.023 (0.965–1.086)	0.445	1.087 (0.969–1.221)	0.156
Gender (male/female)	0.033 (0.000–4.833)	0.180	0.000 (0.000–Inf)	0.973
Pathologic stage	1.868 (0.982–3.552 )	0.057	7.133 (0.064–79.317)	0.110
Tumor stage	0.869 (0.427–1.768)	0.698	0.206 (0.035–1.218)	0.081
Node stage (N-/N+)	3.105 (1.077–8.953)	0.036	1.043 (0.184–5.908)	0.962
Metastasis stage (M-/M+)	3.431 (0.948–12.413)	0.060	0.004 (0.000–1.544)	0.069
Histologic grade	0.869 (0.418–1.807)	0.707	0.330 (0.088–1.234)	0.099
Prognostic score	1.090 (1.041–1.141)	<0.001	1.091 (1.034–1.15)	0.001

LncRNAs play an important role in a variety of biological processes (Kornienko et al., 2013). Accumulating evidence, suggesting that lncRNAs influence the expression of target gene by regulating the transcription and stability of target gene (Batista & Chang, 2013; Tripathi et al., 2013). LncRNA-mRNA co-expression network is an important way to analyze the function and regulation mechanism from a comprehensive perspective. We proposed a prognosis-related lncRNA-mRNA co-expression network in ESCC consisting of 22 lncRNAs, 40 mRNAs, and 77 interaction pairs. Five prognosis-related hub RNAs (CDCA2, MTBP, CENPE, PBK, AL033384.1) were identified and their prognostic value was verified by K-M plots.

Considering that mRNAs are the implementers of molecular function, GO enrichment analysis revealed that mRNAs in the prognosis-related co-expression RNA network were mainly enriched in cell cycle, mitotic cell cycle and nuclear division. Previous studies have shown that cell cycle pathway played an important role in the occurrence and development of esophageal squamous cell carcinoma (Gao et al., 2014; Sanchez-Vega et al., 2018), our observations were consistent with these results.

However, there were some limitations to this study, which should be considered when interpreting our results. First, in this study, only lncRNA, miRNA, and mRNA with both differential expression and prognostic value were included in the analysis. Therefore, the prognostic scoring system and co-expression network may not represent all molecular features that may be associated with ESCC overall survival. Second, several novel signature molecules with important prognostic significance in ESCC lack *in vivo* or *in vitro* experiments to determine their underlying molecular mechanisms. Finally, another limitation of the study was that the prognostic scoring system was not validated in another independent cohort.

## Conclusions

In brief, we constructed a prognostic scoring system based on multiple types of RNA for ESCC that showed high predicting prognosis performance, and deeply understood the regulatory mechanism of prognosis-related lncRNA-mRNA co-expression network. These findings provide promising clues for effective prediction of clinical outcomes.

##  Supplemental Information

10.7717/peerj.8368/supp-1 Figure S1 Heatmap of differentially expressed top 50 up-regulated and top 50 down-regulated RNAs between ESCC and normal tissues (A) lncRNAs; (B)miRNAs; (C) mRNAs.

10.7717/peerj.8368/supp-2 Table S1 The list of differentially expressed lncRNA, miRNA and mRNA

10.7717/peerj.8368/supp-3 Table S2 Univariate Cox regression analysis results of lncRNA, miRNA and mRNA of the remaining 80 ESCC samples

10.7717/peerj.8368/supp-4 Table S3 The correlation results of prognostic lncRNA and mRNA analyzed by Spearman method, and the absolute value of correlation coefficients ≥ 0.4 and *p* < 0.05

10.7717/peerj.8368/supp-5 Table S4 Gene ontology analyses of the mRNAs in prognosis-related co-expression RNA network

10.7717/peerj.8368/supp-6 Table S5 lncRNA-based prognostic cox model parameters, miRNA-based prognostic cox model parameters and mRNA-based prognostic cox model parameters

10.7717/peerj.8368/supp-7 Table S6 Prognostic Cox model parameters based on multiple types of RNA

10.7717/peerj.8368/supp-8 Table S7 Normalized expression matrix of lncRNA

10.7717/peerj.8368/supp-9 Table S8 Normalized expression matrix of miRNA

10.7717/peerj.8368/supp-10 Table S9 Normalized expression matrix of mRNA

10.7717/peerj.8368/supp-11 Table S10 Raw expression matrix of lncRNA

10.7717/peerj.8368/supp-12 Table S11 Raw expression matrix of miRNA

10.7717/peerj.8368/supp-13 Table S12 Raw expression matrix of mRNA

## Additional Information and Declarations

### Data Availability

The following information was supplied regarding data availability: The clinical datasets are available from TCGA (TCGA_ESCA) and the expression profile of esophageal squamous cell carcinoma data are available in the Supplemental Tables.
