# Supplementary figures and images for: Genome-wide analysis of lncRNAs, miRNAs, and mRNAs forming a prognostic scoring system in esophageal squamous cell carcinoma
Source: PeerJ. 2020 Feb 10;8:e8368. doi: 10.7717/peerj.8368 (PMC7017795; doi:10.7717/peerj.8368)

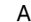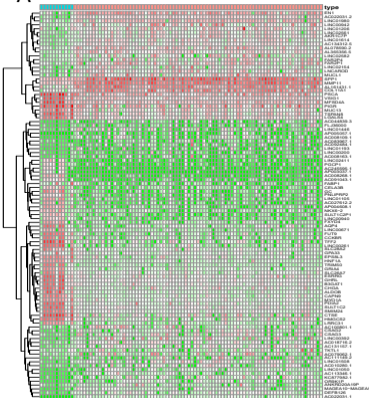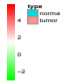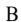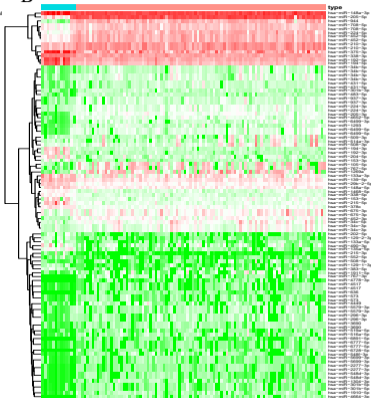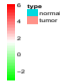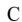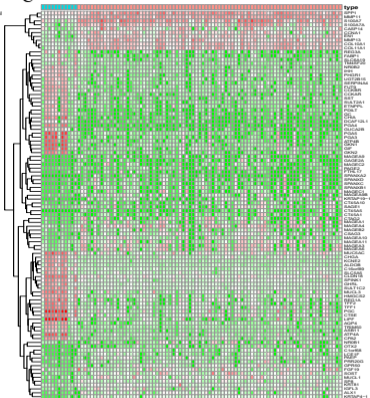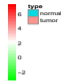

Supplement: Figure S1 — (A) lncRNAs; (B)miRNAs; (C) mRNAs. [file peerj-08-8368-s001.pdf]
